# *In vitro* alteration of physiological parameters do not hamper the growth of human multipotent vascular wall-mesenchymal stem cells

**DOI:** 10.3389/fcell.2015.00036

**Published:** 2015-06-04

**Authors:** Carmen Ciavarella, Silvia Fittipaldi, Silvia Pedrini, Francesco Vasuri, Enrico Gallitto, Antonio Freyrie, Andrea Stella, Elena Gostjeva, Gianandrea Pasquinelli

**Affiliations:** ^1^Laboratory of Clinic Pathology, Department of Specialty, Diagnostic and Experimental Medicine (DIMES), S. Orsola-Malpighi Hospital, University of BolognaBologna, Italy; ^2^Laboratory of Metakaryotic Biology, Department of Biological Engineering, Massachusetts Institute of TechnologyCambridge, MA, USA; ^3^Unit of Surgical Pathology, Department of Specialty, Diagnostic and Experimental Medicine (DIMES), S. Orsola-Malpighi Hospital, University of BolognaBologna, Italy; ^4^Unit of Vascular Surgery, Department of Specialty, Diagnostic and Experimental Medicine (DIMES), S. Orsola-Malpighi Hospital, University of BolognaBologna, Italy

**Keywords:** MSCs, survival, hypoxia, dry culture, hypothermia, MMP-9, anhydrobiosis

## Abstract

**Background:** Mesenchymal stem cells (MSCs) with multilineage potential and anti-inflammatory property can be isolated from different human tissues, representing promising candidates in regenerative medicine. Despite the common criteria of characterization, many factors contribute to MSC heterogeneity (i.e., tissue origin, coexistence of cell subsets at different stage of differentiation, epigenetic) and no standard methods have been approved to characterize MSCs in cell culture.

**Aim:** The present study aimed to test whether MSCs resist adverse chemical and physical culture conditions, surviving MSC subpopulations are endowed with the stemness abilities; to characterize MMP expression in AAA-MSCs under the adverse experimental conditions.

**Methods and Results:** MSCs enzymatically isolated from human abdominal aortic aneurysm (AAA-MSCs) were exposed to media acidification, hypoxia, starving, drying and hypothermia through the following strategies: (1) low-density seeding in closed flasks; (2) exposure to a chemical hypoxia inducer, cobalt chloride; (3) exposure to a dry environment with growing medium deprivation and culture at 4°C. None of these conditions affected MSC viability and stemness profile, as evidenced by NANOG, OCT-4, and SOX-2 mRNA expression in surviving cells. A significant MMP-9 decrease, especially when AAA-MSCs were exposed to hypothermia, was associated with stress resistant stem cells.

**Conclusions:** AAA-MSCs survive to extremely adverse culture conditions, keeping their morphology and stemness features. Besides MMP-9 role in pathological tissue remodeling, this protease may be related to MSC survival. Future studies on MSCs derived from other tissues will be necessary to refine our culture protocol, which can represent an empirical method to demonstrate MSC stemness, with potential implications for their clinical use.

## Introduction

The concept of “cell” originated in 1667 from Hooke's findings and the first attempt to cultivate cells from animal tissues was conducted in 1885 by Roux, who isolated the chick medullary plate and kept it alive for some days in saline solution. Two years later, Arnold cultivated the first leucocytes and many cell culture studies followed (White, 1954). First recorded *in-vitro* functional cell line was cultured in 1955 by Ted Puck and named HeLa (Puck et al., 1956). Since then, there is a large choice of commercial cell line selection in many repository: about 4000 cell lines are maintained by the American Type Culture Collection (ATCC). Currently, many laboratories routinely isolate and grow differentiated as well as stem cells from healthy and pathological tissues to establish primary cell cultures.

Our research group, in the last 15 years, succeeded in establishing viable cell lines with characteristics of mesenchymal stem cells (MSCs) from different vascular tissues. The vascular wall-MSC (VW-MCSs) are negative for CD45 and co-express CD44, CD90, and CD105 molecules, like the bone marrow-derived MSCs (Pasquinelli et al., 2007). Further, VW-MSCs express the neuronal stem cell intermediate filament, nestin, the stemness markers stromal precursor antigen-1 (Stro-1), sex-determining region Y-box-2 (SOX-2 or SRY), neurogenic locus notch homolog protein-1 (Notch-1) and octamer binding transcription factor-4 (Oct-4), while maintaining the ability to differentiate into mesengenic as well as vascular, i.e., endothelial and leiomyogenic, lineages, when cultured in appropriate induction media (Pasquinelli et al., 2010; Valente et al., 2014).

A review of the literature indicates that 130 years after stem cell discovery, the specificity of stem cell antigenic profile is still debated (Vasuri et al., 2014) and the identification of stem cells in culture is even more elusive due to the lack of a standard and coherent methodology to select them in culture (Hart, 2014).

Usually stem cells, that are believed to be highly glycolytic (Simsek et al., 2010), are expanded in closed systems (i.e., flasks) with glucose-bicarbonate buffered media as Eagle's MEM, at 37°C, 5% CO_2_; they are seeded at a proper density unit that is chosen by taking into account the cell dimensions, the growth kinetics and the flask surface.

Conversely, some sporadic studies have proven that both glucose and CO_2_ have detrimental effects on stem cell growth: glucose induces mesenchymal/endothelial stem cell senescence and apoptosis, while the glucose level reduction increases stem cell proliferation and colony forming activity (Kränkel et al., 2005; Saki et al., 2013). In 1985, Barngrover modified the L-15 medium, that was tailored by Leibowitz to promote the growth of the less glycolytic cells (Leibovitz, 1963), by substituting galactose with fructose; this allowed to maintain an optimal pH and lactate/pyruvate ratio (Barngrover et al., 1985). Similarly, cell culture is conventionally performed at 5% CO_2_ and 21% oxygen (O_2_ atmospheric concentration; 151.2 mm Hg): these gas concentrations are far away from those measured *in vivo*; according to Souza (2007), oxygen levels range from 1% (7.2 mm Hg) in the bone marrow tissue to 12% in lungs (86.4 mm Hg). Accordingly, CO_2_ and O_2_ diffusion in flask strongly influences cells growth; low O_2_ tension (i.e., quasi-physiological) stimulates human embryonic cell growth and cellular differentiation as well as low density seeding (Balin et al., 1984). Also hypothermia (4°C) was shown to affect stem cell behavior *in vitro*; hypothermia was used to select satellite muscle stem cells vs. fibroblast, and, most importantly, skeletal muscle stem cells remained in a dormancy state in human cadavers stored at 4°C for 2 weeks giving origin to myotubes after that *ex vivo* cultures have been established (Latil et al., 2012; Marg et al., 2014). These spotted observations indicate that stem cells may survive to a range of stressful conditions including low temperature, starving and hypoxia; even more interesting is the evidence that here we have given about the possibility of the stem cells to survive in an anhydrobiotic dry environment, i.e., in flasks without culture medium or any nutrient supplement.

In this paper the experimental adverse culture conditions described above were empirically replicated in our laboratory using a MSC population isolated from aortic aneurysm (AAA-MSCs), a human cell model characterized by expression of mesenchymal and stemness markers as well as matrix metalloproteinase-9 (MMP-9) upregulation (Ciavarella et al., 2015).

This study was aimed (i) to test extreme culture conditions in a model of MSCs isolated from abdominal aortic aneurysm (AAA-MSCs) (ii) to test AAA-MSC ability to survive to hypoxia, starving, anhydrobiosis and hypothermia, (iii) to analyze AAA-MSC transcriptional profile regarding stemness factors and MMPs.

## Materials and methods

### Isolation and culture of human MSCs

AAA-MSCs were isolated from the abdominal aorta of three male patients who underwent surgical repair for aneurysm, after obtaining Local Ethic Committee Approval (APP-13-01). Aortic tissues were provided by the Vascular Surgery Unit, S. Orsola—Malpighi University Hospital (Bologna, Italy). AAA-MSC isolation and characterization were performed as previously described (Valente et al., 2014; Ciavarella et al., 2015). Briefly, 2 cm^2^ sections of aneurysmal wall were enzymatically digested with 0.3 mg/mL Liberase type II (Liberase TM Research Grade, Roche) in serum-free Dulbecco's modified Eagle's medium (DMEM; Sigma Aldrich) at 37°C o/n in a rotor apparatus. The tissue homogenate was filtered through cell strainers of different size (100-70-40 μm) and centrifuged at 1200 rpm. Cell viability was assessed by Trypan Blue exclusion. AAA-MSCs at passage 0 were cultured in DMEM enriched with 20% Fetal Bovine Serum (FBS; SIGMA Aldrich) at 37°C under an atmosphere of 5% CO_2_ and expanded *in vitro*.

The transcriptional profile of stemness genes, and MMP-2, MMP-9, was performed to evaluate whether the extreme culture protocols we set up could affect AAA-MSC gene expression.

**Figure 1:**
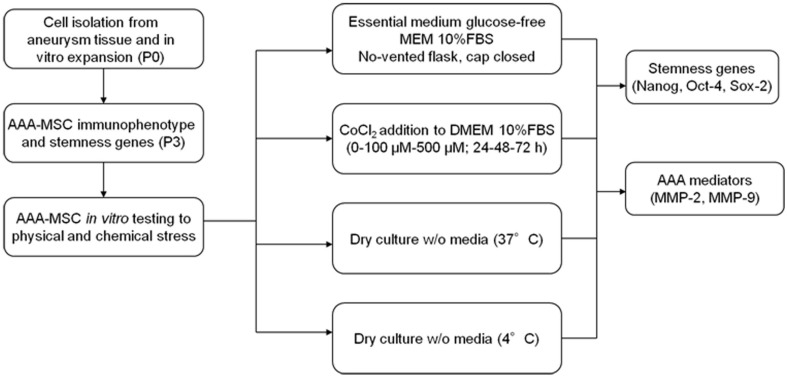
**Experimental design**.

AAA-MSCs at passage 3 were tested for their ability to survive in stressful *in vitro* conditions, consisting in the lack of oxygen and nutrients, through different unconventional strategies. As explained in the experimental design (Figure 1), these strategies included cell culture in closed flasks, using an essential glucose-free medium (MEM) and the complete lack of media either at 4°C or 37°C.

### Low-density culture of AAA-MSCs in minimum essential medium

AAA-MSCs were seeded at low density (200 cells/cm^2^) in no-vented T25 flasks with closed cap to keep the cells in a hypoxic environment.

Cells were grown in 10 ml of Minimum Essential Medium (MEM, Gibco), a specific medium under the intellectual property of Prof. W. Thilly (LIMB, Dept. of Biological Engineering, MIT, Cambridge MA) and manufactured on-demand by Gibco. This medium is free of D-glucose, antibiotics and sodium bicarbonate and contains D-fructose. For *in vitro* analysis we added L-glutamine 4 mM and 10% FBS. Culture media were regularly changed each 10 days. After 3 days from seeding, we measured the pH of surnatant, making a comparison with the cells grown in conventional DMEM. At the end of the experimental protocol AAA-MSCs were processed for RNA extraction.

### Hypoxic culture of AAA-MSCs using CoCl_2_

As an alternative strategy to induce oxygen deprivation, we exposed AAA-MSCs to a chemical inducer of hypoxia, Cobalt (II) Chloride hexahydrate (CoCl_2_, Sigma). CoCl_2_ is a mimetic agent used *in vitro* to induce cellular responses mediated by hypoxia (Piret et al., 2002; Wu and Yonda, 2011).

AAA-MSCs at passage 3 were seeded at a density of 10^4^/well in a 96-well plate in traditional DMEM supplemented with 10% FBS, following the standard cell culture procedures and after 24 h CoCl_2_ was added. The treatment was performed for 24–48–72 h at increasing concentrations (0–100 μM–500 μM). After any treatment, cells were processed for RNA extraction.

Cell viability after CoCl_2_ treatment was estimated by Sulforhodamine B (SRB) assay. After treatment, the cells were washed twice with PBS and fixed in 50% aqueous trichloroacetic acid (TCA) for 1 h at 4°C, rinsed several times with water and incubated with 50 μl/well SRB solution (0.4% in 1% acetic acid) for 30 min. SRB solution in excess was washed off by 1% acetic acid. The cells were incubated in 10 mM Tris for 20 min and the absorbance of each well was measured in a microplate reader (Bio-Rad, Hercules, CA, USA) at 570 nm. The results were expressed as a percentage of treated on controls (untreated cells).

### AAA-MSC dry culture (4°C–37°C)

The third strategy consisted in inducing an anhydrobiotic condition by removing the culture media completely at low temperature. For this purpose, AAA-MSCs were deprived of culture media and kept at 4°C. In parallel, AAA-MSCs from the same sample were treated as described above and maintained at 37°C for 3 days. To ensure the complete environment dehydration, the flasks were housed in a vertical position. After 7 days DMEM 10% FBS was added, flasks were incubated at 37°C and after 14 days treated with trypsin and cells seeded until confluence. At the end of the experimental protocol AAA-MSCs were processed for RNA extraction.

### Total RNA extraction and cDNA synthesis

AAA-MSCs grown in the described culture conditions, were processed for RNA extraction using TRIreagent (TRIzol reagent, Invitrogen, Italy) according to the manufacturer's instructions. One μg of total RNA was reverse transcribed in a 20 μL volume of reaction using High Capacity Reverse Transcription Kit (Applied Biosystems). RNA integrity and concentration were measured using a ND-1000 spectrophotometer (NanoDrop, Fisher Thermo, Wilmington, DE, USA). Reverse transcriptase PCR was performed on all RNA samples with an absorbance (260/280) ratio between 1,8 and 2,2. cDNA was synthesized from 2 μg of total RNA by using High Capacity Reverse Transcription Kit (Life Technologies).

### RT–PCR stemness gene expression analysis

Stemness gene primers are listed in Table 1. The PCR primers were purchased from Invitrogen and SIGMA Aldrich. Glyceraldehyde-3-phosphate dehydrogenase (GAPDH) was used as housekeeping gene to value the cDNA quality. All PCR products were analyzed on 2% agarose gel electrophoresis with Tris-acetate-EDTA buffer 1X, stained with ethidium bromide incorporation and photographed under ultraviolet light. A 100 bp DNA ladder was loaded to allow PCR product size identification. The gel was subjected to electrophoresis at a constant 100 V for 45 min.

**Table 1:** **List of primers used for RT-PCR detection of stem cells transcriptional factors in AAA-MSCs exposed to stressful *in vitro* cultures**.

**Gene**	**Primer sequence**	**Product size (bp)**	**T (°C)**
GAPDH	FWD 5′-ACCACAGTCCATGCCATCAC-3′ REV 5′-TCCACCACCCTGTTGCTGTA-3′	452	61
NANOG	FWD 5′-AAGGCCTCAGCACCTACCTA-3′ REV 5′-ACATTAAGGCCTTCCCCAGC-3′	326	58
OCT-4	FWD a 5′-CTCCTGGAGGGCCAGGAATC-3′	380	62
	FWD b 5′-ATGCATGAGTCAGTGAACAG-3′ REV 5′-CCACATCGGCCTGTGTATAT-3′	402	
SOX-2	FWD 5′ACCGGCGGCAACCAGAAGAACAG-3′ REV 5′-GCGCCGCGGCCGGTATTTAT-3′	208	62

*bp, pair of bases; °C, centigrade degrees*.

### Quantitative real-time polymerase chain reaction

Real Time Polymerase Chain Reaction was performed to investigate the transcriptional levels of MMPs and apoptotic genes in AAA-MSCs exposed to the extreme culture conditions. Real Time PCR analysis was carried out in a Gene Amp 7000 Sequence Detection System (Applied Biosystems) using the TaqMan approach for the β-glucuronidase (GUS), B-cell lymphoma-2 (BCL-2), BCL-2 associated protein (BAX) genes (Applied Biosystems) and SYBR green approach for all other genes, using specific couples of primers, purchased from SIGMA-Aldrich: MMP-2 (FWD 5′-CCCCAAAACGGACAAAGAG-3′, REV 5′-CTTCAGCACAAACAGGTTGC-3, MMP-9 (FWD 5′-GAACCAATCTCACCGACCAG-3′, REV 5′-GCCACCCGAGTGTAACCAT-3′).

Each assay was executed in triplicate and target gene expression was normalized to the housekeeping GUS gene. The final results were determined by the comparative 2^∧^-DDCt method (Livak and Schmittgen, 2001) where DDCT = [CT Target − CT Gus] treated AAA-MSCs -[CT Target − CT Gus] control AAA-MSCs. Results were expressed as fold changes relative to AAA-MSCs grown in standard culture conditions as controls.

### Immunofluorescence assay

Immunofluorescence was performed on AAA-MSCs exposed to CoCl_2_ to detect MMP-9 protein and its alteration following hypoxic induction. Briefly, 4 × 10^4^ AAA-MSCs were cultured on collagen biocoated slide chambers (BD Bioscence, San Jose, CA, USA) and after 24 h CoCl_2_ was added to cultures according to the concentration range used for all the other experiments: 0–100 μM–500 μM for 24, 48, and 72 h. at the end of the treatment, cells were gently were washed with PBS and fixed with cold absolute methanol, for 10 min air dry at room temperature. Fixed cells were then blocked in 1% bovine serum albumin (BSA) in PBS solution and donkey serum, specific for the secondary antibody species, for 30 min at room temperature.

After blocking, cells were incubated with primary antibody anti-MMP-9 (1:500, Cell Signaling) for 1 h at 37°C. Samples were then washed with PBS and incubated with Alexa Fluor 546 (1:250; Invitrogen, Carlsbad, CA, USA) secondary antibody in 1% bovine serum albumin in PBS for 1 h at 37°C in the dark. Finally, after washes, the samples were mounted and nuclei counterstained with Pro Long anti-fade reagent with DAPI (Molecular Probes, Milan, Italy). Images were acquired by a Leica DMI4000 B inverted fluorescence microscope (Leica Microsystems, Milan, Italy) at × 20 magnification.

### Statistical analysis

All experiments were performed in triplicate. Results were analyzed by GraphPad Prism 5 statistical software (GraphPad Software Inc) and are expressed as mean ± standard deviation. Statistical analysis was performed using *t*-test and Two-Way ANOVA test for comparison between more than two groups, followed by Bonferroni *post-hoc* test. Results were considered statistically significant at the 95% confidence level (*p* < 0.05).

## Results

### Properties of MSC isolated from aneurysm wall (n = 4)

AAA-MSCs were obtained from 4 aortic tissues kindly provided by the Vascular Surgery Unit, S. Orsola—Malpighi University Hospital (Bologna, Italy). Clinical data of patients enrolled in this study are described in Table 2.

**Table 2:** **Clinical characteristics of abdominal aortic aneurysm patients**.

**Clinical data of AAA patients (*n* = 4)**	
Age (mean ± S.D. and range)	68.75 ± 5.7 (61–74)
Sex	Males
DAAA (mean ± S.D. and range)	66.25 ± 13 mm (52–70)
Cholesterol (mean ± S.D. and range)	155 ± 28 mg/dl (116–178)
LDL (mean ± S.D. and range)	84 ± 27 mg/dl (49–110)
HDL (mean ± S.D. and range)	51.3 ± 7.8 mg/dl (43–60)
Triglycerides (mean ± S.D. and range)	109 ± 34.28 mg/dl (99–155)
Smoking (%)	75
Hypertension (%)	100
Diabetes (%)	0
Statins (%)	50

*DAAA, abdominal aortic aneurysm diameter; LDL, low density lipoprotein; HDL, high density lipoprotein*.

**Figure 2:**
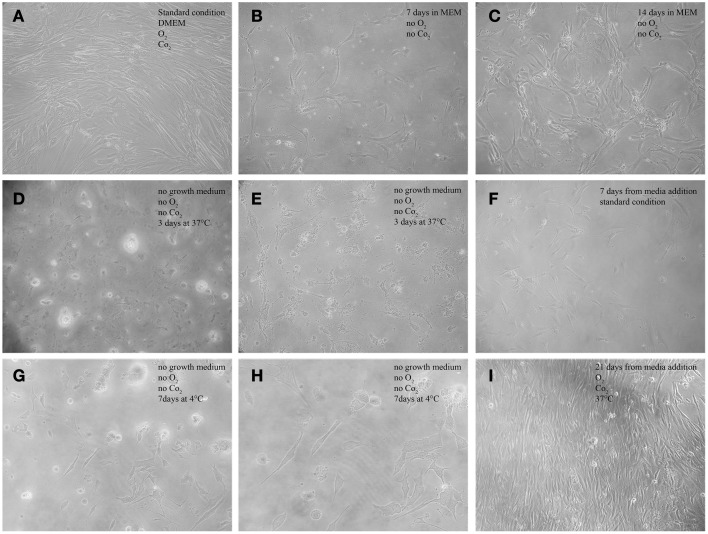
**Morphological features of AAA-MSCs exposed to hypoxia, nutrient deprivation, dry culture, and/or cold temperature. (A)** Typical spindle-shape morphology of confluent AAA-MSCs at passage 3, grown in standard culture conditions; **(B)** Clusters of AAA-MSCs cultured in no-vented closed flasks using MEM glucose-free at 7 days and **(C)** at 14 days from seeding, where a cell monolayer is visible; **(D,E)** AAA-MSCs subjected to media deprivation lost their typical structure, cytoplasmic prolongation and plasma membrane; **(F)** 7 days from media addition, an amount of survival cells was detected; **(G,H)** AAA-MSCs after media deprivation and 7 days at 4°C showed a volume contraction and the typical cell shape was lost; **(I)** after traditional media addition, AAA-MSCs restored their features and reached confluence; (**A–G,I**: 100x magnification; H: 200x magnification).

AAA-MSCs were adherent to plastic substrate, exhibiting a typical spindle-shaped morphology (Figure 2A), expressed mesenchymal markers on their surface and stemness genes Oct-4, Nanog, SOX-2. A 400-fold increased MMP-9 expression both at the mRNA and the protein level was observed in AAA-MSCs when compared to MSCs isolated from healthy aorta (Ciavarella et al., 2015).

### AAA-MSCs are able to survive under different stress culture conditions

AAA-MSCs grown in hypoxic condition and in glucose-free medium showed a reduction of the supernatant pH at day 3, compared to AAA-MSCs derived from the same aortic sample and cultured according to the standard procedures (pH 6.4 in MEM vs. pH 7.5 in the control). The pH acidification did not influence the cell viability, indeed we noticed clusters of surviving AAA-MSCs at day 7 (Figure 2B) and after 2 weeks a monolayer of cells was detectable; no sign of cell death or suffering was simultaneously observed (Figure 2C). Thus, the AAA-MSCs demonstrated high resistance to acid environment and glucose deprivation.

Oxygen deprivation was also achieved through the use of CoCl_2_ that was added to AAA-MSCs cultures in traditional DMEM with 10% FBS. Interestingly, after 24 h exposure to 100 μM CoCl_2_ we did not observe any change in cell viability as measured through the Trypan blue exclusion assay. This result demonstrated a higher cell resistance to the CoCl_2_ cytotoxic concentration, as documented in the literature (Wu and Yonda, 2011). Sulforhodamine B assay on AAA-MSCs exposed to higher CoCl_2_ concentrations for a longer time, showed a dose- and time-dependent reduced cell viability (**Figure 4A**). A significant decrease of cell growth was observed when AAA-MSCs were exposed to 500 μM CoCl_2_ for 48 h (the relative absorbance in CoCl_2_-treated AAA-MSCs was 0.58, compared to 0.9 in untreated control; *p* < 0.001, Two-Way ANOVA test, followed by Bonferroni *post-hoc* test) and 72 h (the relative absorbance in CoCl_2_-treated AAA-MSCs was 0.63, compared to 0.9 in untreated control; *p* < 0.05, Two-Way ANOVA test, followed by Bonferroni *post-hoc* test).

The third drastic strategy consisted in the complete lack of culture media (dry environment) and the maintenance at low temperature. Figure 2 shows the morphological aspect of AAA-MSCs after culture media discard: cells encountered a volume reduction and lost their typical morphology (Figures 2D,E, 37°C; Figures 2G,H 4°C). When traditional media was added, cells were cultured according to the standard procedures, and after two passages AAA-MSCs that have restored their original phenotype were observed (Figures 2F,I). Hypothermia was more effective than the 37°C alternative in selecting highly stress resistant AAA-MSCs: after 7 days at 4°C, cell viability was strongly decreased, as no cells were detected in the flask under the light microscope. After passaging the cells in culture, we could notice an expanded colony of 8 cm^2^, suggesting that only few cells could survive and be expanded at low temperatures.

### Transcriptional profile of AAA-MSCs exposed to stress in *in-vitro* cultures

**Figure 3:**
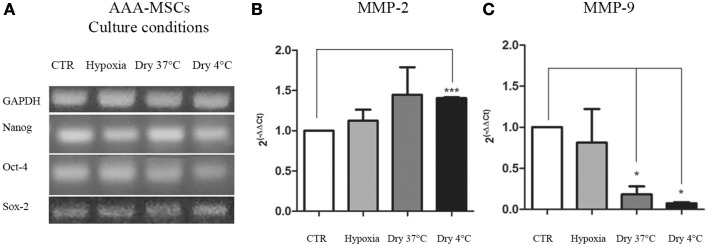
**Transcriptional profile of AAA-MSCs exposed to hypoxia, nutrient deprivation, dry culture and/or cold temperature. (A)** NANOG, OCT-4 and SOX-2 expression was evaluated by RT-PCR and revealed that the oxygen deprivation and the growth medium removal (at 37 and 4°C) did not affect the transcription of genes involved in self-renewal and survival processes. GAPDH was used as housekeeping gene. Results are representative of three independent experiments. **(B)** MMP-2 and **(C)** MMP-9 mRNA expression in AAA-MSCs exposed to the described culture protocols. A significant increase of MMP-2 transcript expression was observed only in AAA-MSCs grown in absence of media at cold temperature; MMP-9 levels of expression decreased in all the experimental conditions, especially when cells were subjected to a dry environment at 4°C. β-glucuronidase gene was used as housekeeping. Results are expressed as fold changes relative to AAA-MSCs grown according to the standard culture protocol (DMEM 20% FBS, 37°C, vented flask). Values are represented as mean ± standard deviation and are representative of at least three independent experiments carried out in triplicate. (^*^p < 0.05; ^***^p < 0.001 paired *t*-test).

As previously shown, AAA-MSCs express transcriptional factors involved in the survival and self-renewal programs typical of stem cells. Here we evaluated the expression of stemness genes NANOG, OCT-4 and SOX-2 after AAA-MSCs have been exposed to the culture conditions described above. No differences in the molecular expression of NANOG, OCT-4 and SOX-2 were observed at RT-PCR in AAA-MSCs exposed to low oxygen levels, media removal and low temperature (Figure 3A); the stemness profile of AAA-MSCs was not affected by the adverse culture conditions.

### Transcriptional analysis of MMP-2 and MMP-9 in AAA-MSCs exposed to stress in *in-vitro* cultures

After the exposure to the extreme culture conditions, AAA-MSCs were processed to evaluate the mRNA production of the AAA molecular mediators, MMP-2 and MMP-9, the latter having been found 400-fold up-regulated in AAA-MSCs when compared to MSCs isolated from healthy control aorta. MMP-2 mRNA showed a weak increase in AAA-MSCs following hypoxia (0.8-fold increased, *p* > 0.05, paired *t*-test) and nutrient deprivation at 37°C (0.7-fold increased, *p* > 0.05, paired *t*-test), in comparison to AAA-MSCs grown according to the standard protocol, but no significant differences were observed; a significant increase was recorded after nutrient deprivation and culture at 4°C (0.7-fold higher compared to controls, *p* < 0.05, paired *t*-test) (Figure 3B). A significant decrease of MMP-9 was observed in AAA-MSCs cultured in a dry environment both at 37°C (5.5-fold decreased in comparison to control AAA-MSCs, *p* = 0.03, paired *t*-test) and 4°C cultures (13.6-fold decreased in comparison to control AAA-MSCs, *p* = 0.02, paired *t*-test) (Figure 3C).

### Molecular profile of AAA-MSCs exposed to CoCl_2_–induced hypoxia

**Figure 4:**
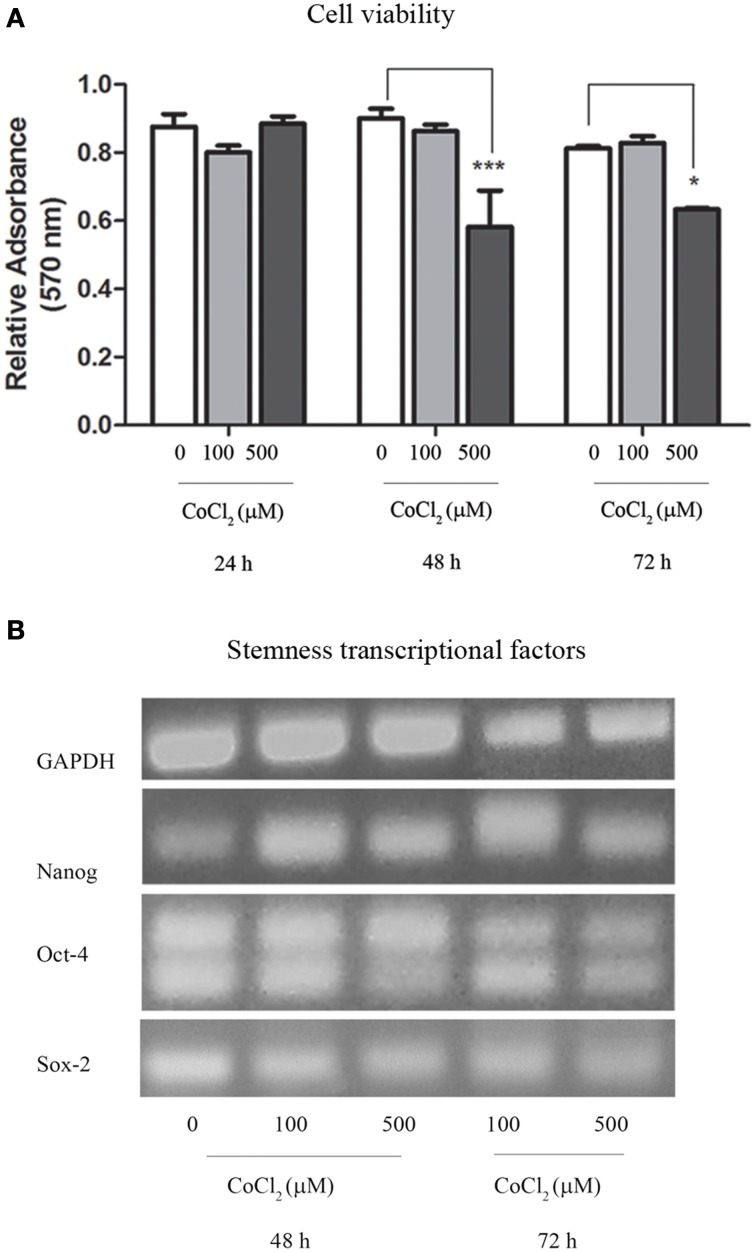
**Cytotoxic assay and transcriptional profile of AAA-MSCs exposed to CoCl_2_. (A)** AAA-MSC viability after 24–48–72 h exposure to CoCl_2_ (0-100-500 μM), measured by sulforhodamine B assay. Values are represented as mean ± standard deviation and are representative of at least three independent experiments (^*^p < 0.05; ^***^p < 0.001; Two-Way ANOVA test, followed by Bonferroni post-hoc test). **(B)** mRNA expression of NANOG, OCT-4 and SOX-2 after 48–72 h exposure to CoCl_2_ (0-100-500 μM), performed by RT-PCR. GAPDH was used as housekeeping gene. Results are representative of three independent experiments.

RT-PCR revealed that stemness genes NANOG, OCT-4, and SOX-2 were expressed in AAA-MSCs grown in the hypoxic environment induced by cobalt chloride (Figure 4B), thus confirming that hypoxia did not influence their stemness genetic profile.

**Figure 5:**
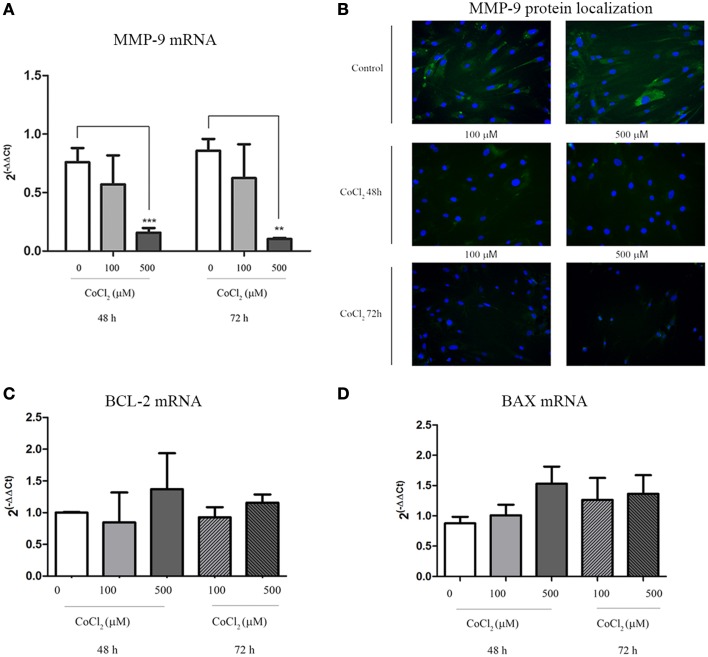
**MMP-9 and BCL-2/BAX expression in AAA-MSCs after CoCl_2_ treatment. (A)** MMP-9 mRNA analysis on AAA-MSCs after 48–72 h exposure to CoCl_2_ (0-100-500 μM) evaluated by Real-Time PCR. **(B)** Immunofluorescence micrographs representing MMP-9 (green) expression treated with CoCl_2_. In untreated AAA-MSCs the MMP-9 protein was revealed in the cell cytoplasm. Following exposure to CoCl_2_ the MMP-9 protein was rarely detectable in cell cytoplasm, especially after 72 h treatment. Nuclei (blue) were counterstained with DAPI. **(C)** BCL-2 and **(D)** BAX mRNA analysis on AAA-MSCs after 48–72 h exposure to CoCl_2_ (0–100-500 μM) evaluated by Real-Time PCR. β-glucuronidase gene was used as housekeeping. Results are expressed as fold changes relative to untreated AAA-MSCs. Values are represented as mean ± standard deviation and are representative of at least three independent experiments carried out in triplicate. (^*^p < 0.01; ^***^p < 0.001 paired *t*-test).

A significant decrease of MMP-9 transcription levels was observed when AAA-MSCs were exposed to CoCl_2_ for 48 h and 72 h (0.65 after 48 h and 0.75 after 72 h-fold decreased, compared to the untreated AAA-MSCs) (Figure 5A). In addition, MMP-9 protein decrease was demonstrated by immunofluorescence detection on AAA-MSCs. Almost no signal was detected in cells treated with 500 μM CoCl_2_ for 48 and 72 h (Figure 5B).

Moreover no significant changes in mRNA expression of BCL-2 and BAX were observed between MSC control and MSCs treated with CoCl_2_, meaning no increase of death cell percentage (Figures 5C,D).

## Discussion

In the present study we demonstrate that mesenchymal stem cells (MSCs) can grow in a non-conventional manner, survive when exposed to extreme adverse conditions, exhibiting high resistance to media acidification, glucose, oxygen, and nutrient deprivation, dry culture, and hypothermia while keeping their morphology and stemness features.

MSCs obtained from human tissues are of great interest for regenerative medicine, thanks to their self-renewal property, multilineage potential (Conget and Minguell, 1999; Pittenger et al., 1999), and immunoregolatory abilities (Ryan et al., 2005).

In 2006, Dominici et al., defined the minimal criteria for MSC characterization: adherence to plastic, specific surface antigen expression, differentiation into adipocytes, osteoblasts and chondroblasts following specific *in vitro* stimulation (Dominici et al., 2006). MSCs can be isolated from various tissues, including adipose tissue (Zuk et al., 2002), umbilical cord blood (Erices et al., 2000), skeletal muscle (Asakura et al., 2001), synovial membrane (De Bari et al., 2001), arterial wall (Pasquinelli et al., 2007). This factor implies that MSCs of different tissue origins show high variability; in addition, many subsets of cells at distinct stage of their differentiation and commitment coexist in the same MSC pool (Karystinou et al., 2009), increasing the MSC heterogeneity and making the MSC application for clinical use difficult.

We investigated a tissue culture model of MSCs isolated from human abdominal aorta with aneurysm disease and assayed three unconventional approaches to culture them, consisting of hypoxia, starving and cold temperature. Developing these conditions generally require specific facilities and, in our laboratory, it has been executed through empirical approaches to easily reproduce them. These methods are supposed to select the stemness compartment of the AAA-MSC population, targeting the surviving cell population that have the highest resistance to stressful conditions, typical features of stem cells at an undifferentiated status.

In the first condition, AAA-MSCs were cultured at low density in an essential glucose-free growth medium, in closed flasks to ensure the O_2_ deprivation. We interestingly noticed that these stressful expedients did not drastically reduce the cell number, despite the supernatant acidification; on the contrary, after 21 days from seeding, AAA-MSCs reached confluence. In addition, we used CoCl_2_, as a chemical inducer of hypoxia, widely applied in hypoxic culture systems. We interestingly observed that AAA-MSCs could resist exposure to CoCl_2_at concentrations that normally result cytotoxic, as shown by the viability assay. A significant cell viability decrease derived only at longer time of exposure (48 and 72 h). These data are consistent with literature indeed hypoxia has been shown to contribute to the undifferentiated status of human MSCs (Basciano et al., 2011).

As hypoxia, both physically and chemically induced, did not influence the AAA-MSC growth, we further investigated the effect of a dry culture in which an anhydrobiotic condition was simulated by the complete removing of the culture media from the flasks that were housed in vertical position at low temperature; the dry culture induces a suffering condition due to the lack of the ordinary nutrient supply, necessary to the cell growing. At the end of the process, we observed the formation of a cell cluster, with a colony aspect, suggesting that clones with high resistance to the extreme environment was selected from the whole cell population. Hypoxia, dry culture as well as hypothermia, did not affect the stemness genes NANOG (Chambers et al., 2003; Hart et al., 2004), OCT-4 and SOX-2 (Richards et al., 2004) expression in our cell model, thus demonstrating that surviving cells express well-established markers of stemness and pluripotency. Surprisingly, MMP-9 was almost undetectable at the mRNA level and a reduced signal intensity was noticed on AAA-MSCx esposed to CoCl_2_ by immunofluorescence assay; this reduced expression was not related to a proportional decrease in cell viability. As recently reported by Ciavarella et al., AAA-MSCs usually present a significant increase of MMP-9 transcript and protein when compared to healthy aortic MSCs (Ciavarella et al., 2015); this increased protein expression was related to the MMP role as molecular mediators of aneurysm pathogenesis. The MMP-9 down regulation in AAA-MSCs exposed to the described protocols, suggest additional functions related to MSC survival and differentiation programs.

Many studies have evaluated the extracellular matrix (ECM) contribution in driving the stem cell fate; MMPs are a family of Ca^2+^ and Zn^2+^ endopeptidases that specifically cleave ECM components (Mannello et al., 2006) and are involved in several pathological processes characterized by matrix remodeling, as well as in cell and tissue development (Vu and Werb, 2000; Nagase et al., 2006; Mannello et al., 2006). MMPs and TIMPs are widely expressed by human MSCs (Silva et al., 2003; Panepucci et al., 2004; Ries et al., 2007), suggesting a crucial role also in self-renewal and/or differentiation (Mannello et al., 2006), although the mechanism is not completely elucidated. Heissig et al. (2002) demonstrated that MMP-9 is tightly associated with hematopoietic stem cell recruitment and mobilization from a quiescent niche to a proliferative microenvironment that allows differentiation, through the release of the soluble kit ligand.

Thus, MMP-9 may be not only involved in vascular and neoplastic disease progression through the ECM degradation, but it can also contribute to define MSC fate, regulating the differentiation and survival program. Future studies exploring the mechanisms involved in MMP-9 modulation will be necessary to elucidate its contribution to stem cell biology.

## Conclusions

We have developed empirical strategies to select a pool of stress surviving MSCs, testing their resistance to extreme conditions, consisting of media acidification, glucose, oxygen, nutrient deprivation, dry culturing and hypothermia. We found that a fraction of MSCs is able to survive, without loosing stemness molecular characteristics. Interestingly, MMP-9 underwent a significant decrease of transcript levels. The hereby proposed protocol confirms that MSCs can grow in physiological-like oxygen concentration conditions, avoiding the use of the abnormally high atmospheric O_2_ concentration and of CO_2_, toxic for human tissues. A further advantage of the proposed culture method is that closed flask limits contact of cells with the external ambient thus preventing bacteria contamination, a frequent event occurring when primary cultures are established. The possibility to grow successfully stem cells in an anhydrobiotic dry environment opens new questions about what are the surviving limits of undifferentiated mammalian cells. Most importantly, this methods do not require any specific or expensive facilities, so it can be performed in any laboratory with basic equipment and it may represent, once further refined, an effective alternative for MSC *in vitro* culture.

### Limits of the study and perspectives

The proposed technique reflects the survival property of a MSC population isolated from a pathological source. Meanwhile, our study presents some limitations.

First of all, these experimental protocols need to be applied to an increased number of samples and tested in cell models representative of different tissue sources, other than vascular wall. The evaluation of stem cell differentiation potential under our conditions constitutes another topic to be investigated.

The MMP-9, beyond its participation to ECM remodeling that occurs during pathological processes, may be correlated to physiologic functions that characterize cell biology, like differentiation, survival and development. We have demonstrated that in a cell model that typically express high levels of MMP-9, the drastic alteration of pH, temperature and nutrient supply causes a significant reduction of its mRNA levels, not ascribed to cell death or suffering. This is a preliminary result that needs to be explored, evaluating what signaling mechanisms are involved.

## Author contributions

CC and SF conceived and designed the experiments, performed the experiments, analyzed the data and wrote the paper. SP designed the experiments, performed the experiments and analyzed the data. FV interpreted data and revised the paper. EG, AS, and AF provided samples, analyzed data and revised paper. EG and GP conceived and designed the experiments, analyzed the data, wrote the paper and revised the paper critically and gave final approval of the version to be published. All authors read and approved the final manuscript.

### Conflict of interest statement

The authors declare that the research was conducted in the absence of any commercial or financial relationships that could be construed as a potential conflict of interest.
